# Antibody Cross-Reactivity in Serodiagnosis of Lyme Disease

**DOI:** 10.3390/antib12040063

**Published:** 2023-10-05

**Authors:** Weronika Grąźlewska, Lucyna Holec-Gąsior

**Affiliations:** Department of Molecular Biotechnology and Microbiology, Faculty of Chemistry, Gdansk University of Technology, 80-233 Gdansk, Poland; wergrazl@student.pg.edu.pl

**Keywords:** *Borrelia burgdorferi* s.l., Lyme disease, serodiagnosis, cross-reactivity

## Abstract

Lyme disease is a tick-borne disease caused by spirochetes belonging to the *Borrelia burgdorferi* sensu lato complex. The disease is characterized by a varied course; therefore, the basis for diagnosis is laboratory methods. Currently, a two-tiered serological test is recommended, using an ELISA as a screening test and a Western blot as a confirmatory test. This approach was introduced due to the relatively high number of false-positive results obtained when using an ELISA alone. However, even this approach has not entirely solved the problem of false-positive results caused by cross-reactive antibodies. Many highly immunogenic *B. burgdorferi* s.l. proteins are recognized nonspecifically by antibodies directed against other pathogens. This also applies to antigens, such as OspC, BmpA, VlsE, and FlaB, i.e., those commonly used in serodiagnostic assays. Cross-reactions can be caused by both bacterial (relapsing fever *Borrelia*, *Treponema pallidum*) and viral (Epstein–Baar virus, Cytomegalovirus) infections. Additionally, a rheumatoid factor has also been shown to nonspecifically recognize *B. burgdorferi* s.l. proteins, resulting in false-positive results. Therefore, it is necessary to carefully interpret the results of serodiagnostic tests so as to avoid overdiagnosis of Lyme disease, which causes unnecessary implementations of strong antibiotic therapies and delays in the correct diagnosis.

## 1. Introduction

Lyme disease is a tick-borne disease caused by bacteria included in the *Borrelia burgdorferi* sensu lato complex (*B. burgdorferi* s.l.). Currently, on the basis of phylogenetic similarity, there are about 21 genospecies within the *B. burgdorferi* s.l complex; however, due to the attempts made to identify and describe new strains, it is believed that this number is not final [1].

Not all bacteria belonging to this group cause borreliosis. In Europe, there are six genospecies causing the disease in humans, i.e., *Borrelia afzelii*, *Borrelia garinii*, *Borrelia bavariensis*, *Borrelia burgdorferi* sensu stricto (s.s.), *Borrelia lusitaniae* and *Borrelia spielmanii*. For many years, it was believed that *Borrelia burgdorferi* s.s. was the only causative genospecies of Lyme disease in North America. However, in recent years there have been more and more reports suggesting that *Borrelia bisettii* and *Borrelia mayonii* may also cause Lyme disease in the USA and Canada [2,3,4].

The vectors transmitting *B. burgdorferi* s.l. are ticks of the genus *Ixodes*, and it is the area of their natural existence that determines the zone of Lyme disease occurrence. There are four main species of ticks that transmit Lyme disease to humans: *Ixodes scapularis* in the eastern United States and Canada; *Ixodes pacificus* in the western United States; *Ixodes ricinus* in Europe and Asia; and *Ixodes persulcatus* found only in Asia [5]. Ticks become infected by *B. burgdorferi* s.l. when feeding on vertebrates that are reservoirs for pathogens. After *B. burgdorferi* s.l. enters the arachnid’s body with blood, the spirochetes localize in its midgut. The increase in temperature and the change in pH inside the tick, accompanying the blood collection, are stimuli for *B. burgdorferi* s.l. to start expressing a new gene pool. This allows the spirochetes to migrate from the midgut to the tick’s salivary glands, from where they enter the body of a new vertebrate host (including humans) together with the saliva [6,7]. 

Lyme disease was first described in the population of Lyme, Connecticut, USA. Rheumatoid-like arthritis was noted in them, which mainly affected children [8]. However, for many years the etiology of this disease was unknown. It was not until the early 1980s that Willy Burgdorfer was the first to discover a new species of spirochete occurring in ticks and link it to observed symptoms [9]. Currently, Lyme disease is the most common tick-borne infection in the northern hemisphere. The increasing number of cases is mainly related to climate change, which leads to an increase in the number of ticks and an extension of their feeding time [10]. The severity of the problem is evidenced by the fact that many countries carry out epidemiological surveillance of Lyme disease [11]. There are approximately 85,000 cases of LB per year in Europe [12]. 

**Figure 1:**
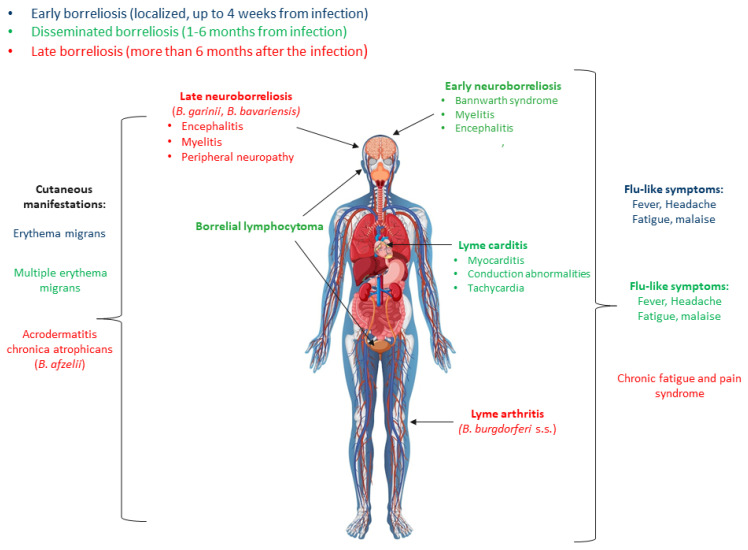
Symptoms of Lyme disease [1,14].

*B. burgdorferi* s.l. does not produce classic toxins or other recognizable virulence factors that directly cause damage to the host tissues. Therefore, it seems that multisystemic disorders are caused by too strong immune reactions to bacterial components [13]. In the course of Lyme disease, three stages are distinguished: early, early-disseminated and late (Figure 1) [1,14,15]. 

The most characteristic manifestation of early Lyme disease is a skin lesion called erythema migrans (EM), which appears in about 60–90% of those infected with *B. burgdorferi* s.l. about 3–30 days after the tick bite [14,16]. At this stage, patients may not show other symptoms or experience flu-like symptoms [17,18]. If the infection is untreated, it can develop into an early disseminated phase after a few weeks or months. The most common disorders include uncharacteristic joint and/or muscle pain and synovitis. In the second stage of the disease, especially in children, early neuroborreliosis may also develop [1,14,15]. One of the possible forms of the disease at this stage is also cardiac disorders, occurring in about 5–10% of patients. A small group of patients may also develop secondary erythema, which, however, is smaller than the primary lesion and is uniformly colored [14,19,20]. The late phase of Lyme disease is a chronic infection that develops from one to several years after infection. Its course is largely dependent on the genospecies that caused the disease, which is probably caused by different tissue tropisms of individual *B. burgdorferi* s.l. representatives [21]. Therefore, some discrepancies in the course of the disease can be observed between Europe and North America and between different regions of Europe. *B. afzelii* is associated almost exclusively with acrodermatitis chronica atrophicans (ACA). *B. garinii* and *B. bavariensis* are often associated with neurological symptoms, and *B. burgdorferi* s.s. mainly causes Lyme arthritis (LA) [22,23,24].

Due to the fact that the symptoms of Lyme disease are not specific enough to allow for a correct diagnosis, laboratory assays play an important role here. As direct methods have many limitations connected with the presence of a very low number of *B. burgdorferi* s.l. in clinical samples and the difficulties in their cultivation, indirect tests based on the detection of class M and G antibodies are most often used [25]. Unfortunately, these tests, due to cross-reactions between the *B. burgdorferi* s.l. proteins used in them and nonspecific antibodies contained in human sera, make diagnostics difficult, leading to overdiagnosis of Lyme disease. This has serious implications for patients who are then subjected to unnecessary strong antibiotic therapy, and the correct diagnosis is delayed, which can lead to catastrophic consequences.

This article presents the problem of cross-reactions in Lyme disease serodiagnostics leading to false-positive results and discusses possible solutions to this problem.

## 2. Characteristic of *B. burgdorferi* s.l.

The *B. burgdorferi* s.l. are microareophilic, Gram-negative bacteria belonging to the type Spirochaetes [26,27]. Due to the presence of the outer and inner cell membranes, these microorganisms are classified as Gram-negative bacteria; however, they differ significantly in structure from typical representatives of this group [28,29]. The following elements are distinguished in the structure of the *B. burgdorferi* s.l. cell: the outer lipid membrane with many surface lipoproteins, the periplasmic space, the flagellum and the inner cytoplasmic membrane forming the protoplasmic cylinder. The flagella and peptidoglycan layer are located in the periplasmic space [28,29].

The complete genome of *B. burgdorferi* s.l. consists of a linear chromosome and numerous circular and linear plasmids. The chromosome mainly encodes the housekeeping genes and its sequence is quite strongly conserved within *B. burgdorferi* s.l. Plasmids carry genetic information responsible for the virulence and pathogenicity [30]. So far, about 21 different plasmids have been identified; however, not all *B. burgdorferi* s.l. isolates have a complete set of them. Only the cp26, cp32 and lp54 plasmids are necessary for the survival of the bacteria in the environment; therefore, they are present in all *B. burgdorferi* s.l. isolates and show a relatively high degree of conservation of the nucleotide sequence. The rest of them have low conservation and are quite easily lost, which is associated with a great difficulty in the laboratory cultivation of fully virulent strains of *B. burgdorferi* s.l. [30,31,32]. 

Due to the complicated structure of the genome, *B. burgdorferi* s.l. are also characterized by a very diverse proteome. The bacteria have the ability to change their surface antigens as an expression of adaptation to changing environmental conditions. Different proteins are present on the surface of the pathogen’s cell during its existence in ticks and vertebrates. The production of proteins necessary for mammalian entry and colonization usually begins in response to changing conditions during a tick’s blood meal. An increase in temperature and a decrease in pH are a signal to start the migration of spirochetes to the salivary glands of arachnids and the production of new proteins needed for transmission [30,33].

Among all *Borrelia* proteins, the best known are surface antigens, which fall into two main categories: lipoproteins, which are anchored to the outer membrane by lipid moieties at the N-terminus, and integral outer membrane proteins (OMPs), which are anchored by transmembrane domains [34]. OMPs are relatively well conserved among *B. burgdorferi* s.l., which is influenced by the location of almost all known genes encoding OMPs on the chromosome. They are mainly responsible for functions necessary for the survival of the bacterial cell, such as obtaining nutrients or resistance to antibiotics [34,35]. Lipoproteins predominate on the surface of the spirochetes, most of which are encoded by plasmids, which leads to a relatively low conservation within *B. burgdorferi* s.l. It is mainly the expression pattern of plasmid-encoded lipoproteins that changes throughout the life cycle of the spirochete in response to changes in environmental conditions. This indicates that surface lipoproteins play an important role in virulence and host–pathogen interactions, and they have also been shown to be involved in host immune response evasion. This opinion is supported by the observation that the loss of plasmids correlates with the decrease in *B. burgdorferi* s.l. infectivity [34,36,37].

A brief description of the most immunogenic *B. burgdorferi* s.l. proteins is provided in Table 1.

## 3. Diagnosis of Lyme Disease

For most bacterial diseases, the direct detection of pathogens via culture is considered the gold standard. Unfortunately, this approach is not used in the diagnosis of Lyme disease due to the very high growth requirements of the spirochetes, the low number of *B. burgdorferi* s.l. in the collected samples and the long waiting time for the result. The above-mentioned limitations mean that this method is mainly used in the case of patients with dysfunctions of the immune system [1,63,64].

Currently, a two-tiered serodiagnostic approach is recommended in the diagnosis of Lyme disease. Due to their high sensitivity and low cost, ELISA tests act as screening tests. However, a positive result does not mean a reliable diagnosis of Lyme disease, as ELISA tests have a fairly low specificity. The Western blot (WB) is used as a second-stage confirmatory test, because, due to the ability to distinguish individual protein bands, it is much more specific. In order to achieve the best diagnostic utility, the most reactive and characteristic antigens produced by *B. burgdorferi* s.l. were selected in the WB test. It has been established that for the detection of immunoglobulin M (IgM) the most useful antigens are OspC (p23–p25), BmpA (p39) and FlaB (p41), and the test is interpreted as positive when there is a reaction to at least two of them [65]. However, for the detection of immunoglobulin G (IgG) the best specificity and sensitivity is obtained taking into account 10 different antigens, i.e., DbpA (p18), OspC (p23–p25), OspD (p28), OspA (p30), BmpA (p39), FlaB (p41), p45, p58, p66 and p93. A test is considered positive when at least 5 of the 10 expected bands appear [66]. Whole-cell lysates (WCLs) of *B. burgdorferi* s.l. are the main source of antigens in both methods.

However, the serodiagnosis of Lyme disease affects several problems; one of them is the serologic window—the time needed for the immune system to produce specific antibodies against pathogens during primary infection. IgMs appear first in the host organism; it takes place about 2 weeks after infection, while the production of IgG usually begins after 3–6 weeks, and they replace IgM as the disease progresses. As the infection continues, the immune response gradually matures so that the sensitivity of serological tests during the later stages of the disease increases significantly [1,67]. 

Another limitation is the great genospecies diversity of *B. burgdorferi* s.l., and the low degree of preservation of amino acid sequences of proteins among them also have a negative impact on the effectiveness of Lyme disease serodiagnostics. This problem is particularly relevant in Europe, where there are as many as six genospecies pathogenic for humans. Most of the immunogenic proteins are heterogeneous, and thus the use of WCL with only one genospecies as antigen preparations in serodiagnosis carries the risk of obtaining a false-negative result [1,32,68,69]. In addition, the diversity of surface proteins is increased by the phenomenon of antigenic variation, which is one way of evading the immune response by *B. burgdorferi* s.l. It is also problematic to obtain WCL containing all of the most immunogenic antigens, as some of them are produced only in vivo [37,70]. The above-mentioned factors cause problems related to the standardization of antigen preparations, which significantly affects the repeatability of the results of diagnostic tests [36,71,72]. Additionally, *B. burgdorferi* s.l. produces many proteins homologous among microorganisms, which carries the risk of cross-reactions and leads to false-positive results in serodiagnostic tests. The lack of specificity of diagnostic tests for Lyme disease prompted the introduction of a two-tiered testing strategy in 1995 [73,74].

The results obtained by the researchers indicate that recombinant proteins or synthetic peptides have the potential to solve some problems affecting the diagnosis of Lyme disease. Careful and thoughtful antigen selection can reduce cross-reactivity and allow test sensitivity to be independent of the *B. burgdorferi* s.l. genospecies that caused the infection, which will simplify the interpretation of diagnostic assays. Recombinant proteins are already used in many commercially available serodiagnostic WB and ELISA tests (e.g., EUROLINE Borrelia-RN-AT (Euroimmun), recomLine Borrelia IgG/IgM (Microgen), Anti-Borrelia plus VlsE (Euroimmun), the ZEUS ELISA Borrelia VlsE1/pepC10 IgG/IgM Test System (ZEUS Scientific, Branchburg, NJ, USA) and the C6 Lyme ELISA kit (Immunetics, Norwood, MA, USA) and displace native *B. burgdorferi* s.l. proteins. 

The most widely used in commercial diagnostic tests are DbpA, OspC, VlsE and synthetic peptides C6 and pepC10. DbpA and VlsE proteins are mainly used in the detection of IgG antibodies. Due to the high variability of DbpA within the *B. burgdorferi* s.l. complex, they are often added in variants from different genospecies for serodiagnostic assays. Thanks to this, it is possible to detect diseases caused by different representatives of *B. burgdorferi* s.l. with one test. C6 is a 25 amino acid peptide derived from VlsE. Its sequence is conserved among *B. burgdorferi* s.l., and therefore C6 is widely used in immunodiagnostic assays [71]. OspC and the 10 amino acid pepC10 obtained from it are mainly used for the detection of IgM in the early stages of infection [75]. 

## 4. Overdiagnosis of Lyme Disease

The specificity of the two-level serological tests in research studies exceeded 99%; however, under these conditions they were performed by highly efficient reference laboratories. Many reports indicate that in real clinical practice two-tiered tests show a much lower specificity [73,74]

Although there are many guides that accurately describe the symptoms of Lyme disease at its various stages and the diagnostic tests that ought to be implemented, these recommendations are often ignored. This results in ordering tests for Lyme disease in cases where there are no clear medical indications for it, which increases the rate of falsely diagnosed Lyme patients. Therefore, serodiagnostic testing should be avoided in individuals who are not at risk of being bitten by a tick or who have only very general symptoms (e.g., pain and fatigue) [76,77]. There are also many problems with the correct interpretation of immunoassays results. Excessive readings of the faint bands in the WB test may be responsible for some false positives. The subjective interpretation of the result is the biggest disadvantage of the WB; it contributes to a low level of reproducibility of the results in various laboratories [78,79].

This is especially true for IgM detection as these are first-line antibodies. Therefore, their presence is often associated with the acute phase of infection. Nevertheless, their reactivity is often nonspecific because IgM is produced when the immune response to the foreign antigen is still immature. IgM should therefore only be used in cases of suspicion of early Lyme disease, i.e., in cases of NB lasting <6 weeks or Lyme carditis (LC). Other clinical symptoms, like LA and ACA, are manifestations of late borreliosis and appear after at least 6 weeks. Therefore, in these cases tests focused on the detection of more specific IgG should be carried out [78].

The usefulness of serological tests is also influenced by the high prevalence of anti-B. burgdorferi s.l. antibodies. This is especially problematic in Europe, as according to a 1995 WHO report the whole continent ought to be considered the endemic area of *B. burgdorferi* s.l. Studies have shown that in some regions of Europe in high-risk groups (hunters, farmers) the prevalence of antibodies against *B. burgdorferi* s.l. antigens is even over 50%, which means that a positive test result does not always mean that the disease is active [76,80,81].

It also turns out that false-positive results may be due to the long duration of the anti-Borrelia antibody in the body. Kalish et al. (2001) studied 79 patients who had been diagnosed with Lyme disease 10–20 years earlier and now showed no signs of active infection. Using a two-tiered test, it was shown that as many as 25% of them had specific IgG and 10% had specific IgM [82]. The *B. burgdorferi* s.l. antigens that were mainly recognized by specific IgM many years after infection were OspC and FlaB. This means that antibodies to proteins such as DbpA (p18), BmpA (p39), p45, p58 and p66 have disappeared over time [82]. However, for IgG in many cases the pattern of recognized antigens did not change even after many years. The most recognizable proteins are DbpA, BmpA, FlaB, p58 and p93 kDa. Patients who developed LA during the first diagnosis showed particular stability in the presence of antibodies, and 62% of them were still positive for IgG and 15% for IgM even after 10 years [82].

## 5. Disorders Most Often Confused with Lyme Disease

There are several infections/diseases that can be misdiagnosed as Lyme disease or vice versa. The most problematic are syphilis, relapsing fever and viral infections caused by Epstein–Barr virus (EBV) and cytomegalovirus (CMV). Furthermore, people positive for rheumatoid factor (RF) are also often misdiagnosed with borreliosis [83,84,85,86,87].

Relapsing fever Borrelia (RFB) are the bacteria closely related to *B. burgdorferi* s.l. This group includes such species as *Borrelia hermsii*, *Borrelia miyamotoi*, *Borrelia hispanica* *Borrelia duttonii* and *Borrelia recurrentis*. These bacteria can be divided into lice-borne fever *Borrelia* (LBRF) and tick-borne relapsing fever *Borrelia* (TBRF). TBRF *Borrelia* are typically transmitted by soft ticks of the genus *Ornithodoros*. Currently, it seems that only *Borrelia miyamotoi* is transmitted by hard-bodied ticks; likewise, *B. burgdorferi* s.l. LBRF is caused only by *Borrelia recurrentis* [88]. TBRF *Borrelia* may be found on all continents except Australia and Antarctica and is a serious public health problem in some parts of the world. LBRF is reported mostly in Africa and single cases in Europe occur mostly among refugees. During the development of relapsing fever, many nonspecific symptoms (e.g., headache, myalgia, chills, nausea, vomiting, arthralgia) and neurological disorders (e.g., meningitis, encephalitis, hemiplegia, facial palsy) may occur. Some of them are similar to the symptoms of Lyme disease, so the clinical differentiation of these two infections is impossible in most cases. It is worth remembering that EM does not appear during relapsing fever, which is one symptom that allows easy and undeniable differentiation of these diseases. In the past, *B. hermsii* whole-cell lysate was used for relapsing fever diagnostics; however, too much antigen similarity between the two groups of bacteria caused a large number of false-positive results. Nowadays, qPCR is mainly used to diagnose relapsing fever. Also, serological tests based on the glycerophosphodiester phosphodiesterase (GlpQ), absent from *B. burgdorferi* s.l., have been developed [85]. 

Syphilis is a sexually transmitted disease caused by spirochetes *Treponema pallidum*. Serological tests for syphilis at all stages of infection remain the mainstay of diagnosis. Antibodies against *T. pallidum* antigens often react with *B. burgdorferi* s.l. proteins, particularly with ELISAs when WCL is used as the source of antigens. This is due to the close phylogenetic relationship of the two pathogens; this cross-reactivity is strongly related to shared flagellar antigens [89]. In the IgM, cross-reactions occur with *B. burgdorferi* s.l. proteins, such as OspC, BBA64, BmpA, FlaB, OspF and OspC, BBA64, BmpA, FlaB and OspF in IgG [86]. So, it may cause false-positive results in immunoenzymatic assays; however, prior infection with *B. burgdorferi* s.l. does not appear to lead to a false-positive syphilis test [90]. It has been proven that the cross-reactions between the antigens of *B. burgdorferi* s.l. and *T. pallidum* can be minimized by incubating the sera with *Reiter treponema* preparations. However, this procedure did not eliminate all nonspecific interactions [91,92].

Human granulocytic anaplasmosis (HGA), formerly known as human granulocytic ehrlichiosis, is caused by *Anaplasma phagocytophilum*, Gram-negative, intracellular bacteria. The main route of transmission of *A. phagocytophilum* is a bite of *Ixodes* ticks. Despite the apparently ubiquitous presence of *A. phagocytophilum* in ticks and animal reservoirs, confirmed clinical cases of HGA in Europe are rare compared to the rest of the world. It has been shown that sera collected from HGA patients show cross-reactions with *B. burgdorferi* s.l. proteins, such as OspC, BBA64, p37, FlaB, VlsE, OspA, OspC and OspF in the M class of antibodies and OspC, p37, FlaB, OspA and OspF in the IgG. Interestingly, despite the detection of these nonspecific antigen–antibody reactions, ELISA tests performed on the WCL did not give false-positive results [86]. However, serum reactivity to both *B. burgdorferi* s.l. and *A. phagocytophilum* antigens is not always due to cross-reactions. People who live in *Ixodes* tick endemic areas and who experience multiple tick bites are probably exposed to the transmission of different pathogens. It is possible that a patient with an HGE infection was previous exposed to *B. burgdorferi* s.l. or vice versa and may produce antibodies to both pathogens. Furthermore, co-infections in humans and vectors have been confirmed [93,94]. Therefore, when HGE, Lyme disease or human babesiosis is suspected, tests for the other tick-born disease should be conducted. Because EM is often absent, clinical manifestations of HGE, such as headache, fever and fatigue, can be confused with those of Lyme borreliosis and other diseases. Laboratory analyses to identify disorders associated with abnormal blood cell counts (i.e., thrombocytopenia or leukopenia) and to determine concentrations of serum hepatic transaminase can help separate HGE from borreliosis [93,95].

People get infected with *Yersinia enterocolitica* and *Yersinia pseudotuberculosis* by ingesting contaminated food or water or from direct infection through blood transfusions. The infection may be asymptomatic, but, in some cases, reactive arthritis similar to LA caused by *B. burgdorferi* s.l. may develop. The most commonly affected joints are the knees and ankles; but other joints, such as those of the toes, fingers, and wrists, may be involved. As in the case of Lyme disease, the diagnosis via cultures is not very sensitive; therefore, serological tests detecting antibodies specific to *Yersinia* antigens may be helpful [96,97]. The newest tests for yersiniosis are focused on detecting antibodies against *Yersinia* outer proteins (YOPs) [98]. Cross-reactivity has been reported between *B. burgdorferi* s.l.-specific antibodies and the YOPs in Western blots. For anti-*Borrelia* IgG, cross-reaction was detected with YopH, YopB, V-ag, YopD, YopN, YopP and YopE, and it was detected for IgA with YopD [96,97]. All *Borrelia* serum samples with this observed cross-reactivity contained IgG against FlaB and specific IgG and IgM against OspC. It may prove that there is antigenic similarity between OspC and FlaB antigens of *B. burgdorferi* s.l. and YopD of *Yersinia* and that two-way cross-reactivity is present. It has been shown that p60, FlaB, OspA and OspC *B. burgdorferi* s.l. antigens are highly cross-reactive with anti-Yersinia sera [96,97]. In this case, therefore, special care should be taken because it is possible to overdiagnosis both Lyme disease and yersiniosis.

Epstein–Barr virus is a gamma herpesvirus causing infection in humans worldwide. The prevalence of EBV is very high; about 90% of adults are infected with EBV. Most infections occur in young children and are asymptomatic or cause nonspecific symptoms [99]. It was observed that serum samples from patients with EBV infection cross-reacted with OspC protein of *B. burgdorferi* s.l. In the studies conducted by Panelius et al. (2002), the percentage of patients with a positive EBV result in the OspC ELISA was initially as high as 73%; however, this cross-reactivity was limited by the addition of sodium thiocyanate and dropped to 46%. Unfortunately, the effect of decreasing nonspecific reactions was not seen with sera specific for syphilis and rheumatoid factor [87].

Cytomegalovirus belongs to viruses in the order *Herpesvirales*. Usually, CMV infection is asymptomatic, but in people whose immune system is defective or immature, such as newborns, people with an overgrowth and patients with AIDS, it can be a serious problem. The virus is highly distributed and the worldwide prevalence of CMV has been estimated at 83% (66–90%) [100]. This means that most people worldwide (similar to EBV) have developed antibodies to CMV, which have been reported to cross-react with *B. burgdorferi* s.l. antigens [83,84,101]. Some commercial ELISAs using *B. burgdorferi* s.l. WCL showed a specificity of only 60% for the detection of antibodies in class M. The low specificity in tests with the use of CMV sera was due to the cross-reactivity of IgM with the OspC and FlaB (p41). In addition, it was shown that anti-CMV IgG recognized such *B. burgdorferi* s.l. antigens as OspC, FlaB, BmpA and VlsE [83,84].

Parvovirus B19 (B19V) is a small, single-stranded, nonenveloped DNA virus. In adults, it can cause joint pain or arthritis with pronounced morning stiffness that may imitate LA caused by *B. burgdorferi* s.l. [102]. Acute B19V infection can induce antibodies that are polyspecific and cross-react with a variety of bacterial antigens, especially for *B. burgdorferi* s.l and other unrelated pathogens, such as *Salmonella* and *Campylobacter*. These antibodies can persist in the circulation for up to 3 months. False-positive results for Lyme disease were obtained not only in the EIA tests but also in the more specific Western blot. Therefore, there is a possibility that an acute B19V infection could be misinterpreted as Lyme disease [103]. One of the likely explanations for this cross-reactivity is polyclonal B-cell stimulation, but the mechanism is not yet fully understood. Another probable mechanism could be molecular mimicry. However, the lack of two-way cross-reactivity (B. burgdorferi s.l.-specific antibodies do not recognize B19V antigens) undermines this theory. Therefore, emphasis should be placed on a sensible interpretation of IgM serological results and the exclusion of recent B19V infection of patients [103]. 

One of the most common symptoms of late borreliosis (especially in the US) is Lyme arthritis. LA shares common clinical features and a synovial histology with rheumatoid arthritis (RA), which means the clinical symptoms are often indistinguishable. Recommended serological tests might also lead to misdiagnosis due to the presence of RF, which is present in the serum of patients with arthritis [104] and other systematic diseases [105]. RFs are antibodies with specificity directed against gamma (γ) globulin and are the most common auto-antibodies ever described in humans [105]. Research has shown that RFs are reactive with many *B. burgdorferi* s.l. antigens, including LA7 (p22), BBA64 (p35), p37, BmpA, OppA2, VlsE and OspC [86,87,106,107]. Additionally, antibodies directed against OspA were detectable in the sera of some RA patients, so it also may be a source of false-positive results of serological tests [104].

Additionally, it was observed that in patients with oral infections cross-reactions with *B. burgdorferi* s.l. antigens, like p37, BmpA, OspF and OspC, occur; however, this phenomenon has not been fully understood [106,108]. 

## 6. *B. burgdorferi* s.l. Cross-Reactive Proteins

The problem of cross-reactions in the serodiagnosis of Lyme disease has been known for a long time. In the early 1990s, a study was carried out to identify cross-reactive *B. burgdorferi* s.l. antigens, trying to increase the specificity of diagnostic tests [109]. Research conducted over the years has focused on the cross-reactivity of the most immunogenic antigens that may have diagnostic usefulness; therefore, mainly surface proteins have been characterized in this respect (Table 2).

OspA and OspB are among the proteins that can lead to false-positive results in serodiagnostic tests for Lyme disease. This is rather surprising, considering that they are produced by *B. burgdorferi* s.l. during their persistence in tick organisms. Nevertheless, numerous studies have demonstrated the presence of antibodies against these proteins in the sera of individuals afflicted with Lyme disease. OspA has even been included as a specific band in the WB reference assays [19,66,85]. It has been showed that antibodies against pathogens such as *A. phagocytophilum*, *T. pallidum*, *Yersinia*, *E. coli* and *B. hermsii* may unspecifically bind OspA and/or OspB [34]. Additionally, OspA appears to be responsible for the autoimmune response of the human body leading to the various disorders. One of them is chronic, antibiotic treatment-resistant Lyme arthritis, which occurs in a subset of patients. It seems likely that this is related to the cross-reactivity of OspA-specific antibodies with human tissues. Similarities in the amino acid sequence were found between OspA (165–173) and leukocyte function-associated antigen 1α, amino acid positions 332 to 340 (LFA-1α 332–340) [111]. Another cross-reacting antigen might be cytokeratin 10, which is present in synovial microvascular endothelium and which cross-reacts with OspA [112]. It has also been observed that anti-OspA IgG levels are directly correlated with the severity and duration of Lyme disease. Furthermore, the analysis of the cDNA sequence obtained from the nervous tissue showed that within it one can find three fragments identical to the amino acid sequences of the OspA protein. Antibodies that are specific to two of the identified fragments have also been shown to react with neurons in the human brain, spinal cord and dorsal root ganglia. Some patients, despite receiving appropriate antibiotics, maintain neurological or cognitive symptoms. It is therefore likely that these symptoms are not caused by persistent infection but by immune-mediated disorders [113].

Another protein with well-described cross-activity is OspC. Although widely applied as an antigen in commercial diagnostic assays for detecting both IgG- and IgM-specific antibodies of *B. burgdorferi* s.l., it has been shown that individuals with various infections and disorders may develop antibodies that nonspecifically bind to the OspC protein [114]. These diseases include HGA, relapsing fever, oral infection, B19, CMV, EBV infection and the presence of RF. One of the sources of these cross-reactions may be C-terminal epitope (PKKP) of OspC. The use of Basic Local Alignment Search Tool (BLAST) analysis identified that this epitope is present in numerous proteins, including two human proteins, such as human tryptase (TPSAB1) and synaptojanin-2 isoform X5 [115]. Moreover, this protein motif was also found in bacteria and edible plants. It is possible to explain that production was initiated by infection with *B. burgdorferi* s.l. and is maintained at a high level by continuous stimulation with cross-reactive autoantigens or with antigens from other microorganisms or environmental factors. Diet can also contribute to this, as the PKKP motif can be found in the glutathione transferase of important crops, such as common wheat and barley [115].

FlaB (p41) shows a high sequence identity of about 40% with flagellin proteins of *Treponema* spp. Especially conserved among these bacteria are the fragments at the ends of the protein (the first 130 and the terminal 70 residues); the central part of the antigen shows higher variability [116]. FlaB shows high reactivity with antibodies, especially in the initial stages of infection, and to exploit the diagnostic potential of this protein, the more specific internal fragment of p41/i, including amino acid residues 129–251, is used [1,117]. However, Luft et al. (1993) showed that a fragment containing amino acids 131–234 is recognized by antibodies contained in the sera of patients suffering from syphilis. So, this region of p41 also contains cross-reactive epitopes [118].

In silico analyses showed that the C6 peptide was similar to domains within the *B. miyamotoi* variable major proteins (Vmps) belonging to the variable large protein (Vlp) family, which are VlsE homologues. *B. miyamotoi* is the etiological factor of TBRF, transmitted by *Ixodes* ticks and likewise *B. burgdorferi* s.l. Cross-reactions of anti-*B. miyamotoi* antibodies against the C6 peptide were confirmed in a study with experimentally infected mice [119]. This cross-reactivity is very problematic because *B. miyamotoi* and *B. burgdorferi* s.l. occupy the same endemic region, so it should be noted that the C6 peptide alone does not allow serological differentiation between the two tick-borne diseases. Therefore, in order to distinguish borreliosis from *B. miyamotoi* infection, a two-stage examination is necessary, along with a critical evaluation of the clinical symptoms [119].

Chandra et al. (2011) reported that about 20% of healthy individuals with no history of Lyme disease had IgG antibodies to p66 [120], which is problematic as p66 band is included in the CDC IgG Western blot diagnostic criteria [73]. These results were confirmed via epitope mapping with the use of microarray technology. This experiment showed that the protein p66 contains numerous linear cross-reactive epitopes with serum samples, regardless of origin or disease state, which were shown to contain antibodies that recognized multiple linear p66 epitopes. These results suggest that its diagnostic utility is questionable and may distort the true test result [56]. BLAST analysis of the peptide sequences did not allow for the identification of any specific proteins in other bacteria that could contribute to the generation of cross-reactive epitopes. However, cross-reactivity can be generated by a significant number of environmental, bacterial, viral or other antigens that are not contained in the sequence databases [56].

In addition, cross-reactivity of *B. burgdorferi* s.l. antigens, such as BmpA, BBA64, OspF, LA7 and OppA2, has also been proven (Table 2). BmpA cross-reacts with antibodies directed against syphilis, relapsing fever, CMV and B19, and it is recognized by rheumatoid factor. LA7 and BBA64 have been shown to be recognized by antibodies contained in the serum of humans suffering from syphilis and HGA and RF-positive patients. Moreover, BBA64 also shows reactivity with anti-*Yersinia* and anti-RF *Borrelia* antibodies. However, research based on the OppA2 antigen, which is not commonly used in the diagnosis of Lyme disease but attracted the attention of scientists due to its production in the early stages of infection, showed that this protein contains linear epitopes recognized nonspecifically by antibodies contained in sera collected from people with syphilis and rheumatoid arthritis [58].

In addition to these antigens, it is also important to remember those highly conserved proteins responsible for the basic metabolism of the *B. burgdorferi* s.l. cell and whose homologs are produced by many other organisms. And although they are not recognized as a marker of Lyme disease by themselves, they can contribute to false-positive results in ELISA tests when WCL is used as an antigen. One example of such a protein is GroEL, a highly conserved heat shock protein, which has a great similarity with proteins for many other bacteria [65,118].

## 7. Conclusions and Future Directions

Lyme disease symptoms (except for EM) are nonspecific, making it impossible to diagnose it properly on the grounds of the clinical picture. For this reason, laboratory methods are the basis for Lyme disease diagnosis, where detection of specific IgM and IgG is the most common [121]. The serodiagnosis of Lyme disease, despite many years of effort, still causes problems. The main reasons are the complicated antigenic structure of the spirochete, the low degree of conservation of protein sequences among genospecies and frequent cross-reactions [1,122].

It is these nonspecific interactions that impose the currently recommended two-tiered algorithm in the serodiagnosis of Lyme disease, increasing the costs of routine diagnosis of Lyme disease and extending the time necessary to make a final diagnosis. It seems that the most problematic in the current approach is the WB, which is time-consuming and difficult to automate. In addition, its reading, as already mentioned, is subjective, which leads to significant discrepancies between laboratories [1,77,78,79]. So far, the problem of Lyme disease cross-reactions has not been studied by many scientists; only a few comprehensive studies have been carried out to identify a wide range of potential disease/pathogen entities causing false-positive test results and to characterize cross-reactive *B. burgdorferi* s.l. proteins [60,83,109,123]. In most cases, such studies focused on single antigens or pathogens, with flagellin [118] and *T. pallidum* [89,90] and *Yersinia* [96,97,124] being the dominant ones, respectively.

Knowledge about cross-reactivity is mainly obtained as complementary information when determining the diagnostic utility of new tests based on recombinant proteins [60,86,108]. Usually, in such studies, few sera containing antibodies against pathogens that are a potential source of cross-reactions are tested. Additionally, these results were obtained using various techniques (WB [96,110], ELISA [60,86,108], immunofluorescence [83], commercial assays [83,107]) and forms of antigens (native antigens [89,110], recombinant proteins [60,86,108]). As a result, information contained in the literature is often difficult to compare and interpret.

It is therefore necessary to conduct comprehensive, wide-ranging research using uniform standards. This will allow for the clear identification of infections/disorders and antigens that may potentially cause cross-reactions in the serological diagnosis of Lyme disease, which will allow diagnosticians to be better prepared to interpret the results. Additionally, it is necessary to follow official recommendations during the diagnostic process. It is important to remember what symptoms are the manifestation of late borreliosis and then perform only detection of highly specific IgG.

Moreover, the creation of new diagnostic tools may contribute to solving this problem. It has been shown that the use of selected recombinant proteins instead of WCL in serodiagnostic tests can significantly reduce the rate of false-positive results [86,108]. Despite the development of genetic engineering for the production of recombinant *B. burgdorferi* s.l. proteins, a specific one-stage assay superior to the standard two-tiered assay has not yet been developed [1,77,78,79]. Enzyme immunoassays based on single recombinant proteins may have low sensitivity as there are hundreds of antigens in the WCL that can be recognized by specific antibodies. By using single proteins, the number of epitopes that interact with immunoglobulins is significantly reduced. Higher immunoassay sensitivity can be achieved through the use of chimeric proteins, which contain selected immunodominant fragments from several proteins in a single amino acid chain. This means that such a protein could be recognized by antibodies specific to several antigens [125,126]. However, in order to show the highest possible diagnostic usefulness, such chimeric proteins must be rationally designed [127,128]. This means that they should be recognized by antibodies against different genospecies of *B. burgdorferi* s.l. and not cross-react with immunoglobulins specific for other antigens.

The easiest and most widely available approach for designing new specific antigens for the diagnosis of Lyme disease is the analysis of amino acid sequences of proteins. Currently, there are a large number of databases containing the amino acid sequences of proteins from many organisms. The most popular are UniProt [129] and NCBI-Protein [130], which contain millions of protein sequences in their collections. When combined with bioinformatics tools like the Protein Basic Local Alignment Search Tool (BLASTp) and ClustalX, these data enable a rapid and straightforward assessment of the conservation level within *B. burgdorferi* s.l. [131,132] proteins and the evaluation of sequence similarity with antigens found in other organisms, thus determining the potential for cross-reactivity. This approach allows for the selection of fragments used for constructing chimeric proteins because it will allow the inclusion of only those fragments conserved within *B. burgdorferi* s.l. and eliminate those commonly found in antigens of other pathogens. Already, in the example of VlsE and FlaB, it has been shown that the proper selection of protein fragments allows for increasing the specificity without reducing the sensitivity of the immunoassay [133,134].

Even greater possibilities in the selection of fragments for the construction of chimeric proteins are provided by B-cell epitope mapping, which allows for detailed knowledge of the distribution of immunodominant fragments and potential cross-reaction fragments [135,136]. There are several methods for identifying conformational and linear epitopes, including both computational and experimental methods [137,138].

Computational methods predict the existence of potential epitopes based on such physicochemical properties as hydrophilicity, solvent accessibility, flexibility, turns, polarity, antigenicity and surface exposure. In general, computational epitope prediction methods can be divided into two groups depending on whether the input is only the amino acid sequence of the antigen or its tertiary structure. These methods using the three-dimensional structure of the protein are characterized by a greater regularity; unfortunately, it is not always possible to use them due to the fact that the spatial structure of many proteins is not known [138]. However, even though in silico methods save time and money, they only allow for the initial identification of potential antigenic determinant sequences. It is advisable to confirm the obtained results using experimental methods [137,139].

The information provided by empirical techniques is much more complete and reliable. Experimental B-cell epitope mappings involve methods such as peptide microarray [140], X-ray crystallography of antigen-antibody complexes [141] and nuclear magnetic resonance [137]. Their main advantage is that they allow the identification of cross-reactive epitopes, thanks to which these fragments can be excluded and not used in the construction of chimeric proteins [135,136,142]. Another aspect is the possibility of distinguishing fragments of antigens that react with different classes of immunoglobulins [143] and thus designing chimeric proteins dedicated to the detection of a specific isotype of antibodies. Therefore, it seems that this approach may be the key to the design of new tools contributing to the improvement of the specificity and sensitivity of Lyme disease serodiagnostic assays, limiting the number of unnecessarily antibiotic therapies and speeding up the correct diagnosis of patients.

## Figures and Tables

**Table 1:** Characteristics of selected proteins of *B. burgdorferi* s.l.

*Bb*sl Life Cycle Stage	Protein	Gene Localization	Information	Refs.
Ticks	OspA (p30)	Plasmid lp54	Essential for the survival of *Bb*sl in the tick A component of the only used Lyme disease vaccine	[38,39]
	OspB	Plasmid lp54	Essential for the survival of *Bb*sl in the tick Transcribed from a common promoter with *ospA*	[38,39]
	OspD (p28)	Plasmid lp38	Binds to the epithelial cells of the tick’s intestine Peak production shortly after feeding and detachment of the tick from the host	[40]
Transmission	OspC (p23–25)	Plasmid cp26	Necessary for *Bb*sl transmission from tick to mammal Production begins when the tick takes a blood meal Binds plasminogen and Salp 15	[21,41,42]
	BBA64 (p35)	Plasmid lp54	Production begins when the tick takes a blood meal Necessary for *Bb*sl transmission from tick to mammal	[43,44]
Mammals	DbpA/B (p18)	Plasmid lp54	Binds decorin and GAGs Involved in the spread and maintenance of the spirochete in the mammalian body	[21,45]
	BBK32	Plasmid lp36	Binds fibronectin, GAGs, and complement component C1r Involved in the spread and maintenance of the spirochete in the mammalian body (vascular and joint colonization) Inhibits the classical pathway of complement	[21,46,47]
	VlsE	Plasmid lp28-1	A *vlsE* locus contains a *vlsE* expression site and 15 silencer cassettes Evades the host’s immune response (antigenic variation) Contains a highly conserved IR6 region	[48,49]
	BmpA (p39)	Chromosome	Binds lamin Highly conserved Necessary for the presence of *Bb*sl in joints, plays a role in the development of LA	[50,51,52]
	OspE-related proteins (OspF, ErpA, ErpC, ErpP)	Plasmid cp32	Binds factor H, Factor H-related proteins and plasminogen Evades the host’s immune response (blocking the complement system)	[53,54,55]
	p66	Chromosome	Integral membrane porin Involved in bacterial dissemination, binds to β3-chain integrins	[56]
	LA7 (p22)	Chromosome	Lipoprotein located mainly in the periplasm Expression increases after *Bb*sl transmission from tick to mammal Genospecies-specific, useful for genotyping	[33,57]
	OppA2	Chromosome	Oligopeptide permease Expressed during early *Bb*sl infection Highly conserved	[58]
Ticks and mammals	FlaA (p37)	Chromosome	Builds a flagellum sheath Production is on a low level	[59,60]
	FlaB (p41)	Chromosome	Builds the flagellum core Highly conserved among a wide range of microorganisms	[61,62]

*Bb*sl—*Borrelia burgdorferi* sensu lato.

**Table 2:** Cross-reactivity of selected *B. burgdorferi* s.l. antigens with IgM and/or IgG.

	WCL	LA7 (p22)	GroEL (p60)	FlaB (p41)	BBA64 (p35)	FlaA (p37)	BmpA (p39)	OppA2	VlsE	pC6	OspA (p30)	OspB	OspC (p23–25)	OspD (p28)	OspE	OspF	Refs.
RFB	IgM+ IgG+	Nd	Rabbit Ig	IgM+ IgG+	IgM− IgG−	IgM+ IgG+	IgM+ IgG+	Nd	IgM+ IgG+	Nd	Rabbit Ig	Rabbit Ig	IgM+ IgG+	Nd	IgM− IgG−	IgM+ IgG+	[86,110]
Syphylis	IgM+ IgG+	IgM+ IgG+	Nd	IgM+ IgG+	IgM+ IgG+	IgM+ IgG+	IgM+ IgG−	IgM/ IgG+	IgM−	Nd	IgM− IgG−	IgM− IgG−	IgM+ IgG−	Nd	IgM− IgG−	IgM+ IgG+	[58,60,86,87,108]
*Yersinia*	Nd	Nd	Nd	IgM+ IgG+	Nd	Nd	IgM+ IgG+	Nd	Nd	Nd	IgM_Nd_ IgG+	Nd	IgM+ IgG+	IgM_Nd_ IgG−	Nd	Nd	[96]
HGA	IgM- IgG-	IgM+ IgG+	Nd	IgM+ IgG+	IgM+ IgG−	IgM+ IgG+	IgM− IgG−	Nd	IgM+ IgG−	Nd	IgM+ IgG+	IgM− IgG−	IgM+ IgG−	Nd	IgM− IgG−	IgM+ IgG+	[86]
EBV	Nd	Nd	Nd	IgM+ IgG_Nd_	Nd	IgM+ IgG+	Nd	Nd	Nd	IgM/IgG+	Nd	Nd	IgM+ IgG+	Nd	Nd	Nd	[60,87,107]
CMV	IgM+ IgG+	Nd	Nd	IgM+ IgG−	Nd	Nd	Nd	Nd	Nd	Nd	Nd	Nd	IgM+ IgG−	Nd	Nd	Nd	[83,84]
B19V	Nd	Nd	Nd	Nd	Nd	Nd	IgM+ IgG_Nd_	Nd	IgM+ IgG_Nd_	Nd	Nd	Nd	Nd	Nd	Nd	Nd	[103]
RA/RF	IgM+ IgG+	IgM+ IgG+	Nd	IgM− IgG−	IgM− IgG+	IgM+ IgG+	IgM+ IgG+	IgM/IgG+	Nd	IgM/IgG+	IgM− IgG−	IgM− IgG−	IgM− IgG−	Nd	IgM+ IgG−	IgM− IgG−	[60,86,87,106,107,108]
HT	Nd	Nd	Nd	Nd	Nd	Nd	Nd	Nd	Nd	Nd	IgM_Nd_ IgG+	Nd	Nd	Nd	Nd	Nd	[109,110]

Explanation of abbreviations: WCL: whole-cell lysates; RFB: relapsing fever *Borrelia*; HGA: human granulocytic anaplasmosis; EBV: Epstein–Barr virus; CMV: cytomegalovirus; B19V: parvovirus B19; RA/RF: rheumatoid arthritis/rheumatoid factor positive; HT: human tissue; IgM+: cross-reactivity with immunoglobulins M; IgG+: cross-reactivity with immunoglobulins G; IgM/IgG+: cross-reactivity with immunoglobulins G and/or M (no differentiation in the reference); IgM−: no cross-reactivity with immunoglobulins M; IgG−: no cross-reactivity with immunoglobulins G; Rabbit Ig: cross-reactive with laboratory-infected rabbits’ immunoglobulins; Nd: no data available; IgM_Nd_: no data available for immunoglobulins M; IgG_Nd_: no data available for immunoglobulins G.

## Data Availability

Data sharing not applicable.
